# Cyclodextrin nanosponge-sensitized enantiodifferentiating photoisomerization of cyclooctene and 1,3-cyclooctadiene

**DOI:** 10.3762/bjoc.8.149

**Published:** 2012-08-16

**Authors:** Wenting Liang, Cheng Yang, Masaki Nishijima, Gaku Fukuhara, Tadashi Mori, Andrea Mele, Franca Castiglione, Fabrizio Caldera, Francesco Trotta, Yoshihisa Inoue

**Affiliations:** 1 Department of Applied Chemistry and Center for Advanced Science and Innovation, Osaka University, 2-1 Yamada-oka, Suita 565-0871, Japan; 2 Department of Chemistry, Materials and Chemical Engineering “G. Natta” – Politecnico di Milano, Piazza L. Da Vinci 32, 20133 Milano, Italy; 3 Department of Chemistry, University of Torino, Via P. Giuria 7, 10125 Torino, Italy

**Keywords:** cyclodextrins, 1,3-cyclooctadiene, cyclooctene, nanosponge, photochirogenesis, photoisomerization

## Abstract

Enantiodifferentiating geometrical photoisomerizations of (*Z*)-cyclooctene and (*Z,Z*)-1,3-cyclooctadiene were performed by using the pyromellitate-linked cyclodextrin network polymer, termed “cyclodextrin nanosponge (CDNS)”, as a supramolecular sensitizing host. The photochirogenic behavior of the nanosponges incorporating β- or γ-cyclodextrin was significantly different from that reported for the conventional sensitizer-appended monomeric cyclodextrins, affording chiral (*E*)-cyclooctene and (*E,Z*)-cyclooctadiene in enantiomeric excesses critically dependent on the solution pH and solvent composition employed, revealing the active roles of chiral void spaces of CDNS in the photochirogenic reaction.

## Introduction

The precise control of chiral photoreactions, or photochirogenesis, is one of the most challenging topics in current photochemistry [1–3]. Weak intermolecular interactions, short lifetime and high reactivity of the excited-state substrate are the major causes that prevent efficient asymmetric induction in chiral photochemistry. A supramolecular approach to photochirogenesis provides a convenient and also promising tool to facilitate the excited-state chirality transfer from chiral host to prochiral substrate through the long-lasting intimate supramolecular contacts of guest substrate(s) with the chiral host in both the ground and excited states [4–10]. Various types of chiral supramolecular hosts, including modified zeolites [11], hydrogen-bonding templates [12], cyclodextrins [13–21] and serum albumins [22–23], have hitherto been employed to mediate chiral photoreactions. External factors, such as temperature [13], solvent [17], pressure [18] and irradiation wavelength [24], have also been found to play crucial roles in controlling supramolecular photochirogenesis.

**Scheme 1:**
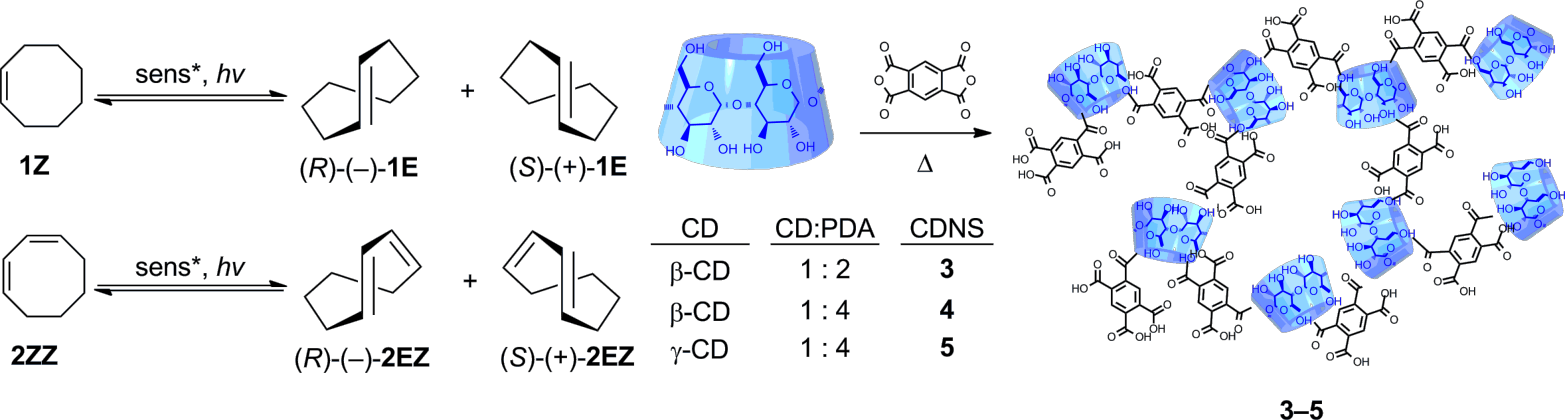
Enantiodifferentiating photoisomerizations of **1Z** and **2ZZ** sensitized by β- and γ-cyclodextrin nanosponges (CDNSs) cross linked by pyromellitic dianhydride (PDA).

**Scheme 2:**
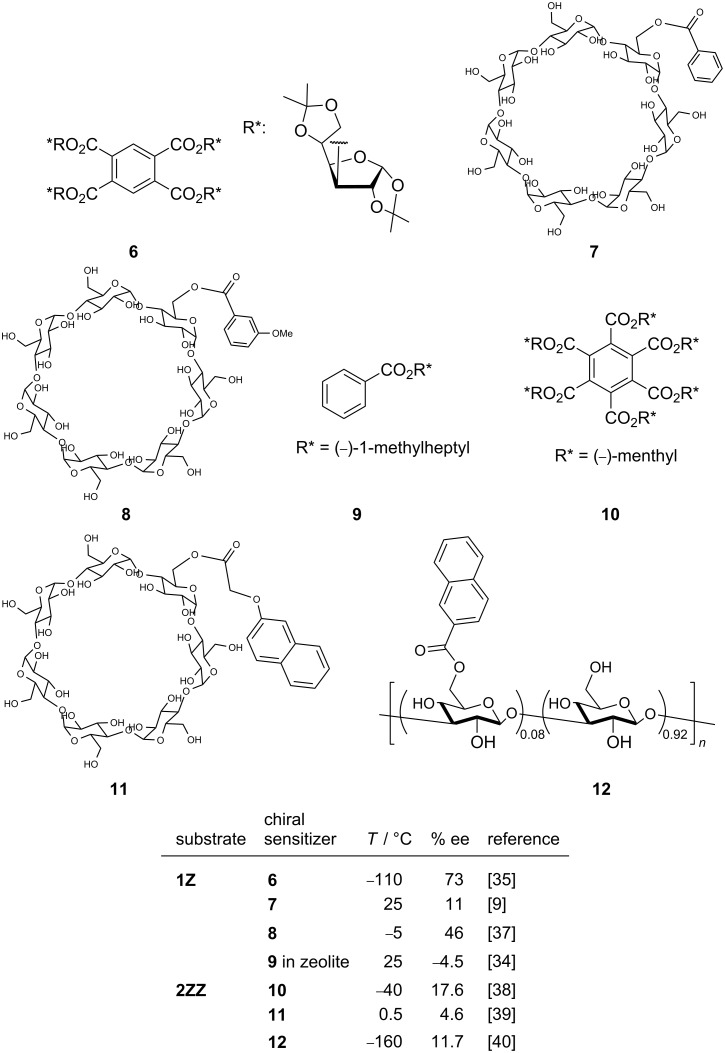
Representative enantiodifferentiating photosensitization of **1Z** and **2ZZ** with conventional and supramolecular photosensitizers.

Amongst the chiral supramolecular hosts that have been applied to photochirogenesis, cyclodextrin (CD) is undoubtedly the most frequently employed, probably due to its ready availability, modifiability, inherently chiral cavity, and optical transparency down to the UV region [3–4]. Nevertheless, the foregoing studies on CD-based supramolecular photochirogenesis have focused primarily on the use of native and singly or doubly modified monomeric CDs. In this communication, we report the results of the first supramolecular photochirogenesis to use pyromellitate-linked polymeric β- and γ-CDs, termed “cyclodextrin nanosponges” (CDNSs) [25–33], as sensitizing hosts for the enantiodifferentiating photoisomerization of (*Z*)-cyclooctene (**1Z**) [34–37] and (*Z,Z*)-1,3-cyclooctadiene (**2ZZ**) [38–40] (Scheme 1). Enantiodifferentiating photosensitization of **1Z** and **2ZZ** was extensively studied by using different kinds of conventional and supramolecular chiral photosensitizers (Scheme 2). The enantioselectivity obtained is generally low to moderate, despite that fact that some reactions were performed at very low temperatures (below −100 °C).

## Results and Discussion

CDNSs **3**, **4** and **5** were prepared in almost quantitative yields by reacting pyromellitic dianhydride (PDA) with β-CD or γ-CD in the presence of triethylamine in dimethyl sulfoxide at room temperature (Scheme 1) and purified by extensive Soxhlet extraction with acetone, as reported previously [41]. We termed these cross-linked CD network polymers as “nanosponges”, because these CD-based polymers show a nanoporous structure and a property of swelling upon absorption of water. The ability to entrap organic molecules can be ascribed to both the pores generated by the polymerization reaction and to the CD cavities. Recently, the relationship between the networking properties of CD nanosponges and the CD/cross linker ratio employed upon synthesis, was revealed by inelastic light-scattering experiments. Thus, the CD/cross linker ratio can be conveniently used as a descriptor of the degree of cross linking and the elastic properties [42]. By using Raman and Brillouin scattering experiments, we found that increasing the PMA/CD ratio leads to an increase in the degree of cross linking, with the frequency of the maximum boson peak in cross-polarized Raman spectra shifting to higher wavenumbers. On the other hand, the stiffness of the polymeric network of CDNSs is more affected by the PMA/CD ratio, rather than the type of CD used.

The adsorption, absorption and inclusion properties of these new materials have been demonstrated for a large variety of substrates, including some pharmaceuticals [25], enzymes [26], pollutants [27], polymers [28], agrochemicals [29], and metal ions [30].

**Figure 1:**
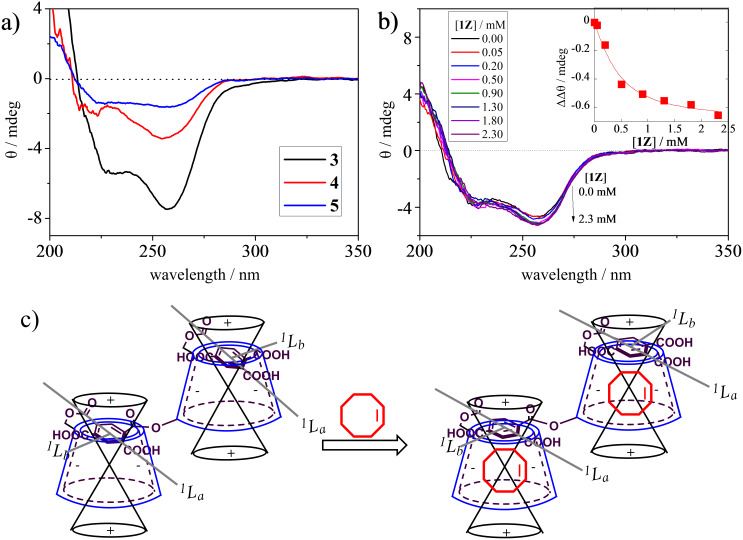
(a) Circular dichroism spectra of **3** (67 μg/mL) (black), **4** (67 μg/mL) (red) and **5** (50 μg/mL) (blue) in pure water at 25 ºC, and (b) of **3** (560 μg/mL) in the presence of 0–2.3 mM **1Z**; Inset shows the nonlinear least-squares curve fit of the ellipticity changes at 257 nm, assuming the 1:1 stoichiometry, from which the apparent binding constant was determined to be 4000 ± 1000 M^−1^. (c) Schematic illustrations of the conformational change of CDNS upon inclusion of **1Z**.

The complexation behavior of **1Z** and **2ZZ** with CDNSs was examined by means of circular dichroism spectroscopy, showing negative induced circular dichroism (ICD) at the ^1^*L*_a_ band and more weakly at the ^1^*L*_b_ band in aqueous solution (Figure 1a). On the basis of the sector rule proposed by Kajtar et al. [43–44], the pyromellitate (PM) units in the polymer network are deduced to be perching on or shallowly included in the CD cavity, placing the ^1^*L*_a_ and ^1^*L*_b_ transitions in the negative region (Figure 1c). The addition of **1Z** to the solution of **3** caused a small but steady enhancement of the negative ICD (Figure 1b), which is rationalized by assuming that the PM unit is excluded from the cavity to better accommodate guest **1Z** and hence becomes more parallel to the portal plane, inducing stronger ICD at the ^1^*L*_a_ band (Figure 1c). Assuming the 1:1 stoichiometry, we calculated the apparent binding constant, averaged over the CD units of CDNS, as 4000 M^−1^ in water at 25 °C. This value is smaller than those obtained with the sensitizer-modified CDs reported previously [36], for which the steric hindrance in the network polymer and/or the less efficient complexation inside the nanoparticle of CDNS would be responsible. The circular dichroism spectral changes observed for the more cross-linked CDNSs **4** and **5** upon binding **1Z** or **2ZZ** were too small to allow quantitative evaluation of the binding affinity.

**Figure 2:**
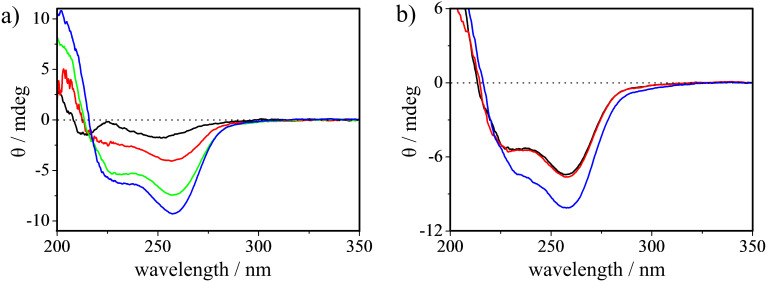
Circular dichroism spectra of **3** (67 μg/mL) (a) in water at pH 1.9 (black), 4.0 (red), 7.5 (green) and 10 (blue) and (b) in water at pH 7.5 (black), in 10% ethanol (red) and in 50% ethanol (blue).

CDNSs **3**–**5** were dissolved in water to make aqueous solutions with concentrations of 45–60 μM in terms of the monomer unit, which were apparently clear at these concentrations but should be a suspension of swollen polymer. In view of the excess amount of PDA used in the preparation of CDNSs, it is likely that some of the carboxyl groups in PDA are not incorporated in the polymer chain, and hence, the solution pH may change the ionic state of the remaining carboxyl groups, affecting the sensitizer conformation and also the product selectivity. The conformational change of the PM units with pH was examined by circular dichroism spectroscopy. As shown in Figure 2a, increasing the solution pH caused a significant enhancement of the negative ICD of CDNS **3**. This seems reasonable, as the ionization of the carboxylic acid moieties on the PM units (p*K*_a1_ = 2.93 for pyromellitic acid; p*K*_a1_ = 2.98 and p*K*_a2_ = 5.28 for phthalic acid) [45] will drive out the shallowly included PM from the cavity but still keep it near the portal with the short ester linker, orienting the ^1^*L*_a_ transition in the more negative region of the sector rule (parallel to the portal plane).

Photoisomerizations of **1Z** and **2ZZ** sensitized by CDNSs were performed at 254 nm under nitrogen in aqueous solutions at different pH values by using a xenon lamp fitted with a band-pass filter (Asahi Spectra MAX-301). As shown in Table 1, the photosensitizations of **1Z** and **2ZZ** with CDNSs gave **1E** and **2EZ** in modest enantioselectivities. Interestingly, the enantiomeric excess (ee) was a critical function of the solution pH. For instance, **3** gave (+)-**1E** in 4.4% ee at pH 1.9 but a doubled 9.0% ee at pH 10. The largest pH-induced ee change was observed for **5**, which afforded (−)-**1E** in 4.8% ee at pH 6.5 but antipodal (+)-**1E** in 9.8% at pH 10. Similar ee behavior was observed upon photoisomerization of **2ZZ** sensitized by **5**, showing a significant increase from 1.0% ee at pH 1.9–6.5 to 7.4% ee at pH 10. These results nicely coincide with the conformational changes of PM units caused by altering the solution pH, providing us with a convenient and also powerful tool for manipulating the stereochemical outcomes of supramolecular photochirogenesis.

**Table 1:** Enantiodifferentiating photoisomerization of **1Z** and **2ZZ** sensitized by CDNSs in aqueous solution at different pH values.

guest	host	pH	*E*/*Z*	% ee

**1Z**	**3**^a^	1.9	0.03	+4.4
		4.0	0.02	+4.5
		7.5	0.04	+7.5
		10	0.03	+9.0
	**4**^a^	1.9	0.03	−0.1
		4.0	0.05	+1.4
		5.2	0.07	+2.1
		10	0.01	+4.3
	**5**^a^	1.9	0.06	+0.1
		4.0	0.09	+0.6
		6.5	0.06	−4.8
		10	0.01	+9.8
**2ZZ**	**3**^a^	1.9	0.04	+5.0
		4.0	0.04	+1.7
		7.5	0.03	+4.6
		10	0.01	+2.7
	**4**^a^	1.9	0.02	+4.0
		4.0	0.07	+1.0
		5.2	0.03	+4.9
		10	0.02	+4.9
	**5**^a^	1.9	0.03	+1.0
		4.0	0.07	+1.0
		6.5	0.13	+0.7
		10	0.01	+7.4

^a^[**1Z**] = [**2ZZ**] = 1.5 mM; [CDNS] = 0.2 mg/mL; irradiated for 1 h at 254 nm in aqueous buffer solutions of pH 1.9–10.0 at 0.5 °C.

It should be noted that γ-CD-based nanosponge **5** gave the highest ee values for both **1E** and **2EZ**. This is in sharp contrast to the result reported for the photosensitization with conventional sensitizer-modified CDs [9,39], in which the more size-matched β-CD, rather than the larger-sized γ-CD, sensitizers consistently afforded **1E** and **2EZ** in (much) higher ee’s. This unusual behavior observed for β- and γ-CDNSs implies the operation of photosensitization and/or enantiodifferentiation mechanisms differing from those proposed for the sensitizer-modified monomeric CDs [9,39]. A plausible alternative mechanism, available for the cross-linked CD polymers, is the photosensitization in a chiral void space surrounded by CD and PM units.

In order to obtain experimental support for the alternative mechanism, we examined the solvent effects on the chromophore conformation of CDNS by circular dichroism spectroscopy and also on the CDNS-sensitized photoisomerization. Indeed, the ICD intensity was enhanced upon the addition of ethanol to an aqueous solution of CDNS (Figure 2b), which encouraged us to further investigate the effect of solvent composition on the photoisomerization. As shown in Table 2, the addition of 10% methanol or 5% ethanol enhanced the product’s ee from 7.5 to 9.8 or 11.7%, respectively, upon sensitization with **3**, while the further addition of methanol (up to 50%) or ethanol (up to 10%) led to less pronounced enhancements. In contrast, the ee of **1E** obtained with the more heavily cross-linked **4** and **5** was not greatly affected or even reduced at high alcohol contents. Crucially, such ee enhancement caused by increasing alcohol content has never been observed for the supramolecular photoisomerization mediated by conventional sensitizer-modified CDs [9], since the addition of alcohol simply reduces the guest affinity [46–47] and hence the ee of the product. This unusual solvent effect upon the addition of alcohol to the aqueous solution of CDNS reinforces the above hypothesis that the enantiodifferentiating photosensitization occurs not in the CD cavity but in the chiral polymer void of CDNS.

**Table 2:** Enantiodifferentiating photoisomerization of **1Z** sensitized by CDNSs in water containing methanol or ethanol.

sens*	solvent	*E*/*Z*	% ee

**3**^a^	H_2_O	0.04	+7.5
	5% MeOH	0.07	+7.8
	10% MeOH	0.02	+9.3
	25% MeOH	0.04	+9.0
	50% MeOH	0.04	+8.5
	5% EtOH	0.06	+11.7
	10% EtOH	0.05	+9.8
**4**^a^	H_2_O	0.07	+2.7
	10% MeOH	0.04	+2.8
	15% MeOH	0.05	+3.2
	25% MeOH	0.02	+1.5
**5**^a^	H_2_O	0.06	−4.8
	10% MeOH	0.02	−4.5
	25% MeOH	0.01	−1.6

^a^[**1Z**] = 1.5 mM; [CDNS] = 0.2 mg/mL; irradiated for 30 min at 254 nm in distilled water containing a varying amount of methanol or ethanol at 0.5 °C.

## Conclusion

The pyromellitate-linked cyclodextrin nanosponges, employed for the first time as supramolecular reaction media for sensitizing the enantiodifferentiating photoisomerization of **1Z** and **2ZZ**, exhibited unique photochirogenesis behavior significantly different from the conventional sensitizer-modified CDs. Thus, the variation of solution pH and solvent composition enabled us to critically control the stereochemical outcomes, leading to the switching of product chirality and the enhancement of the ee of the product by adding alcohol. The latter result in particular revealed the active roles of chiral voids in CDNS as novel photochirogenic reaction media, encouraging the further application of CDNS to chiral photochemistry.
